# The Role of Selected Elements in Oxidative Stress Protection: Key to Healthy Fertility and Reproduction

**DOI:** 10.3390/ijms25179409

**Published:** 2024-08-29

**Authors:** Marcin Wróblewski, Weronika Wróblewska, Marta Sobiesiak

**Affiliations:** 1 Department of Medical Biology and Biochemistry, Ludwik Rydygier Collegium Medicum in Bydgoszcz, Nicolaus Copernicus University in Torun, 87-100 Torun, Poland; 2 Student Research Club of Medical Biology and Biochemistry, Department of Medical Biology and Biochemistry, Faculty of Medicine, Ludwik Rydygier Collegium Medicum in Bydgoszcz, Nicolaus Copernicus University in Torun, 87-100 Torun, Poland; 316714@stud.umk.pl; 3 Department of Inorganic and Analytical Chemistry, Faculty of Pharmacy, Ludwik Rydygier Collegium Medicum in Bydgoszcz, Nicolaus Copernicus University in Torun, 87-100 Torun, Poland; marta.sobiesiak@cm.umk.pl

**Keywords:** fertility, oxidative stress, reproduction, trace elements

## Abstract

Oxidative stress and its relationship to fertility and reproduction is a topic of interest in medicine, especially in the context of the effects of trace elements and micronutrients. Oxidative stress occurs when there is an excess of free radicals in the body, which can lead to cell and tissue damage. Free radicals are reactive oxygen species (ROS) that can be formed as a result of normal metabolic processes, as well as under the influence of external factors such as environmental pollution, UV radiation, and diet. Oxidative stress has a significant impact on fertility. In men, it can lead to DNA damage in sperm, which can result in reduced semen quality, reduced sperm motility and increased numbers of defective sperm, and free radical damage to sperm cell membranes causing a reduction in the number of available sperm. In women, oxidative stress can affect the quality of female reproductive cells, which can lead to problems with their maturation and with embryo implantation in the uterus and can also affect ovarian function and disrupt hormonal regulation of the menstrual cycle. A proper balance of trace elements and micronutrients is key to protecting against oxidative stress and maintaining reproductive health. Supplementation with appropriate elements such as zinc, selenium, copper, manganese, chromium, and iron can help reduce oxidative stress and improve fertility. This work discusses the effects of selected elements on oxidative stress parameters specifically in terms of fertility and reproduction.

## 1. Introduction

Oxidative stress (OS) occurs when the production of free radicals surpasses the body’s antioxidant defenses. Reactive oxygen species (ROS) such as hydroxyl radicals (^•^OH), superoxide anion radicals (O_2_^•−^), and hydrogen peroxide (H_2_O_2_) are highly reactive molecules that damage cellular components to balance their electron shortage [1,2]. The excessive production of ROS can coincide with an increased formation of reactive nitrogen species (RNS), resulting in oxidative/nitrosative stress, a condition commonly observed in a range of human diseases [3]. To counteract surplus ROS, cells have developed a comprehensive antioxidant defense system comprising both enzymatic antioxidants, such as superoxide dismutase (SOD), catalase (CAT), and glutathione peroxidases (GPx), and non-enzymatic antioxidants [3]. Antioxidants can also be categorized by their origin, including those synthesized endogenously, such as enzymes and small molecules, and those acquired from the diet, such as phenolic compounds, flavonoids, phenolic acids, carotenoids, vitamins, and trace elements (TE) [4]. TE, such as selenium (Se), manganese (Mn), and chromium (Cr), and micro-minerals, such as zinc (Zn), iron (Fe), and copper (Cu), exhibit significant effects on reproductive health. Additionally, selenium, zinc, iron, copper, and manganese can be integral to the function of antioxidant enzymes, playing a vital role in sustaining enzymatic activity, and are also cofactors of many enzymes involved in redox reactions [5,6,7,8]. Infertility has become a growing global health concern over the last twenty years. Supplementing with specific trace elements can help mitigate oxidative damage and improve the chances of successful conception and a healthy pregnancy [9]. A healthy antioxidant system is essential for neutralizing ROS, protecting reproductive cells and tissues, and supporting normal fertility. Despite the known link between oxidative stress and unexplained infertility, effective treatments remain elusive. In this review, we briefly discuss the links between oxidative stress, trace elements, and infertility.

## 2. Methods

We conducted an extensive literature search using the ISI Web of Science/PubMed/Science Direct/Google Scholar databases containing information on the impact of trace elements on oxidative stress in the aspect of reproduction and fertility. The following keywords were used in the data search: (“fertility” and “trace elements”, “selenium”, “zinc”, “copper”, “iron”, “manganese”, “chromium”); (“oxidative stress” and “reproduction” and “selenium”, “zinc”, “copper”, “iron”, “manganese”, “chromium”); (“oxidative stress” and “selenium”, “zinc”, “copper”, “iron”, “manganese”, “chromium”); (“reactive oxygen species” and “fertility” or “reproduction”).There were no restrictions in collecting the data. No language restrictions were applied during the analysis. Rather, we tried to select articles from the last 20 years. After searching, we further examined the full texts of the literature to determine eligibility for inclusion in this review. Editorials, conference abstracts, and studies with incomplete or unavailable data were excluded.

## 3. Reactive Oxygen Species in Reproductive Health

The balance between oxidants and antioxidants is crucial for women’s reproductive health. Elevated levels of ROS are crucial in several stages of the ovulation process, such as cumulus expansion, progesterone synthesis, gene expression before ovulation, and the initiation of ovulatory signals [10,11]. A balanced ROS level in follicular fluid not only reflects healthy follicular metabolism but may also serve as a predictive marker for the success of in vitro fertilization–embryo transfer [10]. Additionally, nitric oxide (^•^NO) plays a vital role in maintaining various reproductive functions, including pregnancy, at normal physiological levels [11]. However, excessive ROS levels can damage cellular structures, impacting the environment of follicular, hydrosalpingeal, and peritoneal fluids, thus affecting egg quality, fertilization, implantation, and early embryo development, leading to oxidative stress-induced infertility [10,11,12]. Fertility declines with age, linked to a decrease in ovarian function, to mitochondrial issues, and to ROS accumulation [12,13]. Furthermore, endometriosis and polycystic ovary syndrome are among the most common diseases associated with infertility, and it has been suggested that they are linked to increased levels of oxidative stress [11,14].

A substantial proportion of infertile men, ranging from 30% to 80%, exhibit elevated levels of seminal ROS, highlighting the significance of assessing seminal oxidative stress in male reproductive health evaluations [7,8]. It is crucial to note that molecules such as O_2_^•−^, H_2_O_2_, and nitric oxide (^•^NO) play pivotal roles at the onset of capacitation, serving as key regulators to promote processes such as cholesterol efflux, cAMP production, and protein tyrosine phosphorylation [15]. ROS contribute to increased membrane fluidity of spermatozoa and enhance the rates of sperm–oocyte fusion, which are critical during the capacitation and acrosome reaction stages [8]. The impact of ROS on capacitation is dose-dependent [15]. While moderate levels are beneficial, excessive ROS activity in sperm may lead to infertility by hindering the sperm’s ability to fuse with oocytes [8]. High levels of ROS can result in sperm dysfunction due to DNA damage, lipid peroxidation, loss of membrane integrity, cell death, diminished antioxidant levels, and ultimately, compromised sperm quality [1]. Sperm cells are particularly susceptible to oxidative stress because of their high polyunsaturated fatty acids content in the membrane and their scarcity of cytoplasmic antioxidant enzymes [1,7]. Mitochondria are a primary source of ROS in cells, with O_2_^•−^ being the principal mitochondrial ROS produced in sperm [16]. The generation of mitochondrial ROS is largely governed by the redox state of the respiratory chain complexes, including proton pumps regulated by the mitochondrial membrane potential (MMP). Maintaining a high MMP is essential for preserving chromatin structure, ensuring normal sperm morphology, promoting high motility, and inducing the acrosome reaction [16]. Excessive ROS production is linked to an increase in sperm subpopulations with low MMP among infertile men, leading to DNA fragmentation, reduced sperm viability, and impaired mitochondrial function, which decreases motility [1,15,16,17]. Another issue affecting male fertility is semen hyperviscosity (SHV). SHV affects male fertility through various mechanisms, including impaired sperm movement, inflammatory responses, oxidative damage, and alteration in levels of trace elements, and poses challenges in assisted reproductive techniques [1]. RNS, a subset of ROS, includes molecules such as peroxynitrite anions and ^•^NO [16]. At normal levels, RNS regulate various sperm functions such as capacitation, acrosomal reaction, and binding to the zona pellucida. Elevated RNS levels can induce nitrosative stress, affecting mitochondrial and sperm function by inhibiting electron transport chain components, altering protein functions through tyrosine nitration and S-glutathionylation, and disrupting energy production, which may lead to apoptosis and necrosis [9,11,16].

## 4. Impact of Trace Elements on Oxidative Stress

Selenium plays a critical role in the body’s antioxidant defense system. In its inorganic form, selenium primarily occurs as Se^4+^ and Se^6+^ [18].Within living organisms, selenium is encountered as selenoproteins (SePs) and possesses antioxidant capabilities. It is integral to the structure of two essential amino acids, selenomethionine and selenocysteine. Selenocysteine is notably present in the catalytic centers of enzymes that exhibit antioxidant functions, such as GPx and thioredoxin reductases [19,20,21,22]. GPx protects cells from oxidative damage by reducing peroxides, harmful by-products of cellular metabolism that can damage cell components such as lipids, proteins, and DNA [23]. A selenium-deficient diet results in reduced GPx activity [24]. Thioredoxin reductase, a significant selenoenzyme, operates alongside NADPH and thioredoxin within the thioredoxin system, offering antioxidant benefits. The thioredoxin system plays a crucial role in mitigating oxidative stress by aiding in the reduction of peroxiredoxin proteins, which eliminate hydrogen peroxide and are oxidized in the process [25]. Selenoprotein P (SePP) functions as a redox protein and affects the redox balance utilizing its thioredoxin domain to distribute selenium necessary for the production of intracellular GPx [26].

Zinc is an essential micronutrient that plays a crucial role in the functioning of approximately 300 metalloenzymes, including oxidoreductases, hydrolases, and ligases [27]. It acts as a cofactor for these enzymes, and a deficiency in zinc can lead to reduced activity of enzymes that depend on it [28]. Zinc also has antioxidant capabilities, effectively blocking the formation of harmful radicals such as hydroxyl radicals (^•^OH) and superoxide radicals (O_2_^•−^) by competing with pro-oxidative metals such as iron (Fe^2+^) and copper (Cu^2+^) [29]. Zinc is also a key component of superoxide dismutase, an essential enzyme that transforms singlet oxygen radicals into hydrogen peroxide [30]. Moreover, it inhibits NADPH oxidases, which are responsible for generating singlet oxygen radicals from oxygen using NADPH [30,31]. Zinc stimulates the production of metallothionein, which neutralizes ROS, and possesses potent anti-inflammatory and antioxidant properties, including inhibition of tumor necrosis factor-alpha (TNF-α)-induced nuclear factor kappa-light-chain-enhancer of activated B cells (NF-κB) activation [30,32].

Iron that is not attached to proteins poses a significant risk to cells by promoting oxidative stress [33]. Iron is an abundant transition metal in food, and in a healthy state, the vast majority of iron within the body is safely incorporated into proteins such as hemoglobin, myoglobin, transferrin, ferritin, and hemosiderin [6,34]. Iron is fundamentally important in hematopoiesis, primarily for its role in hemoglobin synthesis [6]. In biological systems, Fe^2+^ ions and H_2_O_2_ participate in the Fenton reaction, resulting in the formation of highly reactive hydroxyl radicals (^•^OH) and ferric ions (Fe^3+^) [25,35]. Free iron acts as a source of ROS, contrasting with Fe bound to proteins which has antioxidant properties. The heme group, containing Fe^3+^, is the catalytic center of all four polypeptide chains of CAT [36]. CAT is an enzyme belonging to the oxidoreductases class that facilitates the conversion of hydrogen peroxide into water and oxygen [37].

Copper, existing in both Cu^+^ and Cu^2+^ forms, serves as a vital cofactor for numerous enzymes that play a role in electron transfer processes within metabolic pathways [38]. It is known for both its pro-oxidant and antioxidant characteristics [39,40]. Copper participates in a Fenton-like redox reaction that leads to the generation of ROS [6,41]. Exposure to elevated levels of copper significantly decreases glutathione level which is an important cellular antioxidant acting against this trace element toxicity [6]. Copper, alongside zinc, is integral to the functioning of antioxidant enzymes such as SOD-1 and SOD-3 [42,43]. A significant portion of copper found in blood plasma is incorporated into ceruloplasmin, a protein known for its antioxidant capabilities [44]. Classified as an oxidoreductase, ceruloplasmin’s activity is dependent on copper ions [45]. This protein plays a crucial role in maintaining iron balance within the body by catalyzing the oxidation of Fe^2+^ to Fe^3+^ and reduces the generation of ^•^OH [46].

Manganese, particularly as Mn^2+^ ions, is a crucial component of the active sites in numerous enzymes [47]. It acts as a cofactor for various enzymes, such as manganese superoxide dismutase (MnSOD), arginase, glutamine synthetase, phosphoenolpyruvate decarboxylase, and pyruvate carboxylase [48]. MnSOD is found in mitochondria, where it forms the primary defense against oxidative stress produced by mitochondrial respiration [43,49]. This enzyme transforms superoxide anion radicals into hydrogen peroxide and oxygen within the mitochondria. Moreover, manganese can indirectly affect the redox state of iron, copper, and other transition metals, enhancing the production of mitochondrial peroxide (H_2_O_2_) even at physiological levels [48].

Chromium exists mainly in trivalent (Cr^3+^) and hexavalent (Cr^6+^) forms, with trivalent chromium being significantly less toxic than its hexavalent counterpart. However, at high concentrations or depending on its chemical ligands, Cr^3+^ can still be toxic [50]. Inside cells, chromium (VI) reduces to the more stable chromium (III), generating genotoxic intermediates responsible for its mutagenic and carcinogenic effects [8]. The reduction of Cr(VI) also produces various reactive oxygen species, such as hydroxyl radicals, singlet oxygen, and superoxide anions, leading to different types of DNA damage and oxidative stress, which can result in lipid peroxidation, altered cellular redox states, and apoptosis [6,51]. Table 1 summarizes the mechanism of action of selected elements on oxidative stress parameters.

## 5. Trace Elements in Reproductive Health

Selenium is a trace element that is naturally present in Brazil nuts, green vegetables, shiitake and button mushrooms, and various seeds such as young barley seedlings. The Recommended Dietary Allowance for selenium is established at 70 µg/day for men and 55 µg/day for women to maintain optimal health. Nonetheless, these recommended levels are viewed as insufficient by some studies, which propose a minimum daily requirement of 90 µg for adults. The World Health Organization sets the maximum safe intake level at 400 µg/day or 5.1 µmol/day for adults 19 years and older, with intake above this threshold potentially being harmful [54]. Selenium is essential for the activity of selenoproteins that protect against oxidative damage [55]. This micronutrient, known for its antimutagenic properties, helps prevent the transformation of normal cells into cancerous ones. Its protective benefits are largely linked to its incorporation into GPx and thioredoxin reductase, enzymes that are essential for safeguarding DNA and cellular components from oxidative damage [12,56,57]. Functional selenium levels are crucial for thyroid hormone production and DNA formation [57]. These processes significantly affect fertility and reproductive outcomes. Despite limited research, evidence suggests selenium’s critical role in ovarian follicle growth and maturity. Sodium selenite, a form of inorganic selenium, not only enhances the growth of oocytes but also boosts the proliferation rate of theca and granulosa cells [55]. In human studies, low levels of selenium in plasma, follicular fluid, amniotic fluid, or tissues, as well as reduced concentrations or activity of GPx in tissues, have been linked with unexplained infertility, miscarriage, preterm birth, and gestational diabetes [58]. Lower selenium levels in maternal blood are correlated with prolonged attempts to conceive and a 46% higher chance of subfertility, even when accounting for various maternal and paternal factors [57]. Only a limited number of studies have shown a link between female fertility, selenium status, and the activity of selenium-dependent GPx. Women undergoing in vitro fertilization (IVF) or experiencing unexplained infertility had lower selenium levels in their serum and follicular fluid. A reduction in selenium-dependent GPx activity within the follicular fluid has been linked to the lack of oocyte fertilization in women undergoing IVF treatments [59]. In a study exploring the effects of sodium selenite, Abedelahi et al. [49] found that selenium supplementation (with 5 or 10 ng/mL sodium selenite added to the culture medium) significantly reduced ROS-induced oxidative stress and enhanced the total antioxidant capacity (TAC) and GPx activity in derivedfromvitrified and non-vitrified ovarian tissue. The growth rates of follicles, oocytes, and embryos were notably higher in the selenium-supplemented groups. An interesting observation is that conditions such as endometriosis and polycystic ovary syndrome, characterized by an excessive production of ROS, may lead to a significant depletion of selenium due to its antioxidant properties [55].The beneficial effects of selenium are crucial for the growth of testicles, the production of sperm, the movement of spermatozoa, and testosterone secretion [5,54,60]. Increased dietary selenium intake has been correlated with higher sperm counts in the semen of infertile men. Studies on selenium supplementation in infertile men have shown enhancements in testicular antioxidant capability, semen selenium levels, sperm count, sperm shape and movement, and overall fertility [58]. Selenium may offer protection against oxidative DNA damage in human sperm cells [56]. Research has shown that men with normal sperm quality have significantly higher levels of glutathione (GSH) compared to those with sperm defects, with a notable correlation between seminal GSH levels and sperm motility [56]. GSH is a major intracellular antioxidant and has a protective role against cytotoxic aldehydes from lipid peroxidation [56,60]. Additionally, the antioxidant function of selenium may be a key factor in explaining the observed increase in lipid peroxidation among infertile patients. Lower selenium levels in the seminal fluid of infertile men have been associated with higher concentrations of malondialdehyde (MDA) in the same individuals, indicating oxidative stress [56]. MDA, the final product of the oxidation of polyunsaturated fatty acids, is a reliable and widely used biomarker for the assessment of lipid peroxidation [2]. Chiti et al. [61] discovered that dietary selenium supplementation led to a 3.7-fold increase in seminal total antioxidant capacity (TAC) levels and a 2.4-fold reduction in sperm with non-progressive motility. The study also noted that selenium, zinc, iron, copper, manganese, and chromium significantly affected the level of MDA in semen. Yin et al. [62] studied the associations between oxidative stress indicators (MDA, SOD, and GSH) in seminal plasma and the risk of idiopathic oligoasthenoteratozoospermia (OAT) among 148 participants, including 75 cases of idiopathic OAT and 73 controls. They also examined how these associations might be influenced by levels of essential trace elements such as selenium, copper, and iron. There was a negative association between GSH levels and the risk of idiopathic OAT in the high Se group, with no significant association in the low Se group. Subgroup analyses showed a positive association between MDA level and the risk of idiopathic OAT in the high Cu group, with no significant association in the low Cu group. The researchers concluded that there is an association between these markers of OS and OAT risk that may be influenced by Cu and Se levels in seminal plasma. The available literature also includes studies in which no correlation was found between Se content, semen parameters, and oxidative status [60].

Diet is a factor determining the level of zinc in the body, and the main sources of this element are legumes, cereals, and meat products. The recommended daily requirement for zinc is 2–3 mg in adults [63]. Recently, zinc has been identified as a crucial element necessary for the completion of meiosis and egg activation in vitro, as well as for follicle rupture and the completion of meiosis in vivo [64]. Zinc plays an antioxidant role in yak oocytes by reducing levels of ROS. Nonetheless, a substantial decrease in ROS levels, resulting from treatment with high concentrations of Zn, could negatively impact the development of bovine embryos and the viability of human cultured cells. In a laboratory setting, excessively high concentrations of zinc that are not physiological can negatively impact the development of embryos before implantation [64]. Zinc is involved in neutralizing free peroxide radicals as part of superoxide dismutase (Zn-SOD), preventing the oxidation of unsaturated fatty acids, regulating plasma vitamin A levels, and counteracting the effects of harmful metals such as cadmium and lead in the ovaries [12]. Zn-SOD is present in oocytes and pre-implantation embryos throughout their passage in the oviduct [64]. Anchordoquy et al. [65] discovered that the activity of SOD significantly increased following zinc supplementation, a change that was associated with improved embryonic development. According to their in vitro research, zinc supplementation during oocyte maturation enhanced SOD activity in cumulus cells, reducing DNA damage and apoptosis in these cells. As a result, the increased SOD activity helped protect the embryo from oxidative stress through to the blastocyst stage. Xiong et al. [64] investigated the impact of zinc supplementation on the maturation and developmental potential of yak (*Bos grunniens*) oocytes during in vitro maturation. The researchers concluded that zinc supplementation during the in vitro maturation process enhanced the maturation and subsequent development of yak oocytes by increasing GSH and SOD levels and reducing ROS levels in the oocytes, demonstrating a beneficial effect of zinc on oocyte quality and developmental capacity. Polycystic ovary syndrome (PCOS) is a prevalent condition that impedes fertility. Women with PCOS are prone to various metabolic issues, including hormonal imbalances and elevated indicators of inflammation and oxidative stress. Research indicates that taking magnesium and zinc supplements for 12 weeks, as compared to a placebo, can positively impact serum high-sensitivity C-reactive protein (hs-CRP), plasma MDA, TAC, and gene expression of interleukin-1 (IL-1) and TNF-α in women with PCOS. However, this supplementation does not affect ^•^NO, GSH, MDA levels, or expression of interleukin-8 (IL-8), transforming growth factor-beta (TGF-β), and vascular endothelial growth factor (VEGF) [66]. Zinc is involved in production of luteinizing, follicular, and testicular hormones, with Zn levels significantly differing between fertile and infertile men [7,60]. Chiti et al. [61] showed that zinc and iron intake positively impacted sperm viability. Zinc is also known for its antioxidant properties and its role in stabilizing sperm DNA and membranes. An elevated zinc level, in the absence of a specific seminal vesicle zinc-binding agent, may hinder the process of sperm chromatin decondensation, potentially leading to chromatin instability. Poor chromatin packaging or integrity is linked to sperm DNA damage, elevating the risk of infertility and adverse pregnancy outcomes [1]. The role of zinc in supporting antioxidant defense suggests that its reduction could make sperm more susceptible to oxidative damage [60]. Nenkova et al. [60] detected lower levels of zinc in men exhibiting poor sperm quality than in controls (men with normozoospermia). Furthermore, these groups showed higher levels of MDA and lower levels of GSH, indicating the presence of oxidative stress. Atgi et al. [56] conducted a study to analyze the antioxidant content in the seminal plasma of men with varying fertility potential, focusing on GSH, zinc, and MDA. The study included semen samples from fertile men (normozoospermics) and infertile patients divided into three groups based on their infertility issues (asthenozoospermics, oligozoospermics, and teratozoospermics). The concentrations of Zn were higher in normozoospermics compared to the infertile groups. Zn was positively correlated with sperm motility and count. Total GSH (GSHt), reduced GSH (GSHr), and GSSG levels were significantly elevated in normozoospermics compared to the infertile groups, with significant correlations between GSH levels and sperm motility and count. MDA levels were markedly higher in the infertile groups, with negative correlations to sperm motility and concentration and a positive correlation with abnormal sperm morphology. The study concluded that lower levels of semen GSH and deficiencies in trace elements are linked to reduced semen quality, suggesting these may serve as important indirect biomarkers for idiopathic male infertility. The measurement of reduced and oxidized thiol levels, as well as the enzymes associated with them, can serve as a valuable approach for assessing the oxidative/antioxidative balance in clinical and epidemiological studies, particularly in relation to male fertility issues such as asthenospermia. Alsalman et al. [67] explored the impact of zinc supplementation on semen qualities and certain biochemical markers in men with asthenospermia. The asthenospermic men received 440 mg of zinc sulfate daily for three months. Semen analyses were conducted before and after the supplementation period, focusing on the levels of reduced and oxidized thiols, the thiol oxidation-reduction index, and the activity of thiol-dependent enzymes in both the sperm and seminal plasma. The findings indicated that before treatment, men with asthenospermia exhibited significantly higher levels of oxidized thiols and significantly lower levels of reduced thiols, sulfhydryl oxidase, and glutathione peroxidase activities compared to the fertile control group. After zinc supplementation, the levels of oxidized and reduced thiols, along with the activities of thiol-derived enzymes in the asthenospermic patients, normalized, suggesting an improvement in the oxidant/antioxidant balance. However, the specific levels of reduced and oxidized thiols within the sperm did not show significant changes post-treatment.

Iron, another critical micronutrient, is crucial for the synthesis of nucleic acids and proteins, electron transportation, cellular breathing, and the growth and specialization of cells, all of which are essential for the development of sperm and their metabolic functions. However, being a transition metal, iron can readily lose an electron during oxidation processes, becoming a source for the production of ROS [5]. Both a deficiency and an excess of iron can affect fertility. Establishing iron intake recommendations poses challenges due to issues in gauging iron consumption. The World Health Organization classifies iron deficiency as ferritin levels below 15 µg/L. However, there is a proposition to raise this threshold to below 30 µg/L to enhance the sensitivity of this measurement across populations, both with and without diseases [68]. Ferritin levels below 30 µg/L are linked to unexplained infertility and an increased likelihood of recurrent pregnancy loss [68]. Huang et al. [69] found higher levels of iron in men with asthenospermia compared to normal controls, as well as increased levels of MDA, but showed no significant associations between this micronutrient and MDA. They suggested that MDA was not formed in the seminal plasma itself, but it may be produced by the spermatozoa, prostate, or other accessory organs. However, during incubation of human sperm with Fe^2+^ it was demonstrated that elevated iron levels had a negative impact on sperm motility linked to lipid peroxidation [70]. Nenkova et al. [60] observed that in groups exhibiting poor sperm quality, iron levels were found to be elevated compared to the group with normal sperm (normozoospermia). Furthermore, these groups showed higher levels of malondialdehyde and lower levels of glutathione, indicating the presence of oxidative stress. The high MDA and the low tGSH (total glutathione) levels contributed to decreased sperm motility and increased abnormal spermatozoa. In a study involving sixty-five fertile male volunteers from the southern region of Poland, participants were categorized into two groups based on the iron levels in their seminal plasma: low iron levels (Fe-L) and high iron levels (Fe-H). The findings revealed that the Fe-H group had a significantly higher percentage of non-motile sperm compared to the Fe-L group. Additionally, activities of SOD and manganese-SOD, as well as MDA levels, were notably lower in the Fe-H group than in the Fe-L group. Conversely, total oxidant status (TOS) and oxidative stress index (OSI) values were significantly elevated in the Fe-H group. The research concluded that in fertile men, high iron levels may adversely affect sperm motility and exacerbate oxidative stress, as well as alter the levels of certain cytokines in human semen [71]. Iron overload leads to oxidative stress, which damages fats, proteins, and DNA, negatively impacting the production of sperm and their metabolic activities. Elevated iron levels are linked to reduced sperm movement and higher TOS values in the seminal fluid of men with normal fertility. Iron’s role is critical not only in situations of infertility or reduced fertility but also in the general population of men with normal fertility [5,71].

Both severe copper deficiency and excess have been linked to increased production of ROS and cell death stemming from mitochondrial malfunction [43]. Furthermore, minor variations in cellular copper levels, which are not toxic, can affect cell growth or differentiation by altering mitochondrial metabolism. This change can regulate the balance between glycolysis and oxidative phosphorylation, as well as ROS production, thereby creating an oxidative cellular environment [72]. A lack of copper markedly impacts depletion or complete elimination of copper-reliant enzymes in the body, subsequently impeding cellular life functions. Conversely, due to its high reactivity, free copper can initiate the generation of an abundance of free radicals, resulting in significant damage to proteins and DNA [60]. The total amount of copper in normal adults is 50–150 mg; this metal concentration is tissue-dependent, varying between 3 mg (kidneys) and 46 mg (skeleton) (average adult ≈ 70 kg) [72,73]. Foods rich in copper, such as seafood (especially shellfish), organ meats, nuts, seeds, whole grains, lemons, raisins, coconuts, papaya, apples, and drinking water can help maintain adequate levels of this micro-mineral [72,74]. Copper is crucial for processes such as iron metabolism and enzyme reactions in nervous system function, as well as in hematopoietic, bone, and other systems [73]. Its role in energy production, iron metabolism, and cardiovascular health ensures optimal functioning of the body’s systems, creating a favorable environment for conception and a healthy pregnancy [43,72]. Some researchers suggest that a mother’s inability to maintain high Cu and Mn levels may contribute, at least in part, to reproductive health problems [75]. A study by Grieger et al. [57] involving 45 women participating in IVF found that elevated levels of copper in urine correlated with a greater total count of oocytes collected and improved embryo quality. Furthermore, an increase in both urinary copper and zinc levels was linked to the overall number of embryos produced. Current research highlights a noticeable increase in serum copper levels during pregnancy, while copper deficiency is often associated with pregnancy complications and miscarriage. An increase in copper concentration in the blood during pregnancy may suggest an increased demand for this metal due to its key role in embryogenesis and fetal development [75]. The existing literature does not contain any documented associations between oxidative stress, copper, and infertility in human females. In men, maintaining proper copper levels is linked to enhanced sperm quality, underscoring its critical function in male reproductive health. Copper is vital for the creation of male reproductive cells, supporting the structural robustness of sperm and boosting their mobility. This improvement in movement facilitates the sperm’s journey through the female reproductive tract towards egg fertilization [76]. Both an excess and a deficiency of copper can markedly diminish male fertility, affecting a broad range of factors from sperm abnormalities, gonadal health, and hormone production to the distribution of micronutrients such as zinc and iron [76]. Enzymes reliant on copper, including ceruloplasmin, SOD (SOD-1 and SOD-3), metallothioneins, and cytochrome c oxidase, are active throughout all phases of gamete formation. They are also present in the somatic cells within the testes and the somatic cells of the epididymis [76]. Abdul-Rasheed [2] highlighted the direct relationship between copper levels in seminal fluid and SOD activity, noting a significant reduction in copper levels in patients with azoospermia, which may lead to reduced SOD activity. He also noticed that malondialdehyde (MDA) levels showed a significant increase over normal control values in oligozoospermic and azoospermic seminal plasma samples.

Typically, mammalian tissues hold between 0.3 and 3.0 μg of manganese per gram of wet tissue weight. Dietary consumption of food and drinking water usually fulfills the body’s manganese nutritional needs. The estimated safe and adequate daily dietary intakes (ESADDI) of manganese necessary to preserve body manganese stores are 2 to 5 mg/day for adults and 1.5 to 2.0 mg/day for children aged 4 to 6 years [48]. Few studies have examined the effects of Mn exposure on male reproductive health. Chiti et al. [61] showed that higher intake of manganese was associated with a 7.8-fold increase in sperm count, and Lapointe et al. [77] found Mn^2+^ was a potent stimulator of sperm motility through stimulation of adenylate cyclase activity. Bansal and Kaur [78] observed that oxidative stress conditions in human sperm, induced by ferrous ascorbate, nicotine, or a combination of both, led to an increase in TSH and GSSG content alongside a decrease in GSH levels and the redox coefficient. The addition of 0.1 mM Mn^2+^ enhanced the TSH, GSH, and redox ratios, while also lowering GSSG levels. The researchers deduced that Mn^2+^ supplementation helps preserve thiol levels by mitigating oxidative stress.

Chromium exists in all animal tissues, typically ranging from a few to several tens of µg/kg and seldom surpassing 100 micrograms µg/kg [79]. Chromium(III) is involved in the metabolism of carbohydrates, lipids, and proteins [79]. Dietary sources of trivalent chromium are whole grains, fish and shellfish, dry edible beans, and potatoes [80]. Trivalent chromium enhances insulin’s action, but it requires a suitable ligand, such as nicotinic acid, to function biologically [50]. This trace element is often included in dietary supplements [79]. Chromium supplements such as chromium picolinate and niacin-bound chromium(III) are marketed for their potential benefits on blood sugar and lipid levels, body composition, and muscle strength [50].

**Figure 1:**
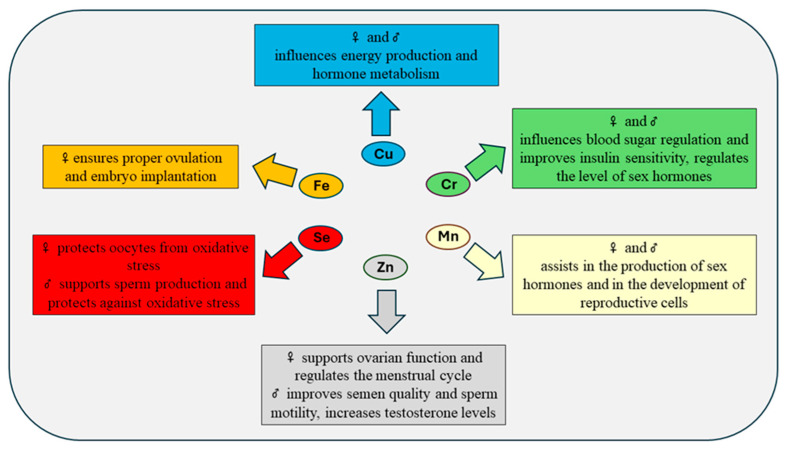
Importance of selected elements on male and female fertility and reproduction (♀—female, ♂—male).

In addition, Amiri Siavashani et al. [81] hypothesized that chromium supplementation might be beneficial in women with PCOS and candidates for IVF who suffer from different metabolic abnormalities. In a randomized, placebo-controlled clinical trial, Jamilian et al. [82] found that administration of chromium (200 μg/day) for PCOS women for 8 weeks resulted in decreased MDA, along with a significant rise in plasma TAC concentrations, in comparison to the placebo. However, chromium supplementation did not significantly impact plasma concentrations of NO and GSH. In another study, a noticeable increase in TAC and a significant reduction in MDA levels, indicating a reduction in oxidative stress, were observed after chromium (200 μg/day) supplementation for 8 weeks in infertile women with PCOS preparing for in vitro fertilization [83]. Trivalent chromium influences male reproductive health by altering levels of ROS, testosterone, and sperm characteristics such as motility and the count of abnormal sperm. The compound can damage Leydig cells, which are crucial for male reproductive health, by blocking enzymes essential for steroid hormone production and triggering mutagenesis and cell death [84]. However, the impact of chromium(III) on oxidative stress in male reproductive organs is still uncertain, with mixed findings on its effects on lipid peroxidation in seminal plasma from numerous studies. Biswas et al. [85] observed a rise in MDA levels in the seminal plasma of male turkeys that received a diet supplemented with chromium picolinate. This increase in MDA levels may indicate heightened lipid peroxidation in the seminal plasma of the turkeys treated with chromium. Conversely, Shanmugan et al. [86] indicated that chromium supplementation did not influence lipid peroxidation of seminal fluid in Dahlem Red peripubertal roosters by measuring levels of MDA. Type 2 diabetes has a considerable impact on male fertility, negatively influencing semen quality by reducing its volume, sperm count, concentration, and motility [84]. In the study conducted by Imanparast et al. [87], 92 patients with type 2 diabetes mellitus received supplements of either chromium picolinate or trivalent chromium along with vitamin D. The research revealed a decrease in MDA levels in the serum after four months, indicating reduced OS. The supplementation notably increased total thiol groups, serving as innate antioxidants, and the combined administration of chromium picolinate and vitamin D significantly boosted the TAC, a critical marker of antioxidant status. This finding supports the antioxidant properties of chromium(III) within cellular systems. Reduced levels of testosterone are a significant factor contributing to male infertility since this hormone plays a vital role in numerous functions across the male reproductive system. Testosterone is synthesized by Leydig cells in the testes through a process known as testicular steroidogenesis. Any disruption in the functioning of Leydig cells can impair steroid production and, consequently, fertility. Endocrine-disrupting chemicals (EDCs), which interfere with hormonal signals, are particularly concerning for their impact on steroidogenesis. Chromium is identified as one of these EDCs [84]. Therefore, although trivalent chromium has been noted for its potential health benefits, its safety remains a contentious issue. However, exposure to chromium(VI) can adversely affect male fertility by causing oxidative stress in the testis, leading to spermatogenesis disruption, structural damage to seminiferous tubules, and decreased antioxidant defenses [8]. The selected elements discussed affect the fertility of both men and women and thus have important implications for reproduction (Figure 1).

## 6. Conclusions

Metal ions such as zinc, copper, selenium, and iron are important in female reproductive health. They are involved in various processes, including follicular development, ovulation, and early embryonic development. The seminal plasma contains various metal ions, including zinc, selenium, copper, iron, manganese, and chromium which play crucial roles in maintaining sperm health and function. Abnormal levels of these metal ions can lead to reproductive disorders, affecting fertility and pregnancy outcomes. Certainly, further research determining the correlations between the content of various components, including trace elements and micronutrients, is needed to get a more complete picture of the effects on many conditions in terms of oxidative stress, including fertility and reproductive problems. 

## Figures and Tables

**Table 1:** Effects of selected elements on oxidative stress parameters.

Element	Impact on Oxidative Stress	Mechanism of Action	Examples of Parameters/ Stressors	Reference
Selenium	Decreases oxidative stress	It is part of the enzyme glutathione peroxidase, which neutralizes lipid peroxides and hydrogen peroxide	Decreased MDA levels, increased GSH levels	[19,20,21,22,23,24,25,26,52]
Zinc	Decreases oxidative stress	Supports the function of antioxidant enzymes such as superoxide dismutase (SOD)	Reduced DNA damage, reduced levels of ROS	[27,28,29,30,31,32]
Copper	Decreases oxidative stress	It is a cofactor of superoxide dismutase (SOD), which neutralizes superoxide anions	Increased SOD activity, decreased in ROS levels	[39,40,41,42,43,44]
Manganese	Decreases oxidative stress	Manganese-dependent superoxide dismutase (MnSOD) cofactor	Decreased MDA levels, increased MnSOD activity	[47,48,49]
Iron	Increases oxidative stress	Participates in the Fenton reaction, which generates hydroxyl radicals	Increased MDA levels, DNA and protein damage	[25,33,34,35,36]
Chromium	Decreases oxidative stress	Trivalent chromium (Cr(III)) is an essential micronutrient that supports glucose and lipid metabolism, which can indirectly reduce oxidative stress	Reduced ROS levels, improved GSH levels	[8,50,51,53]
	Increases oxidative stress	Hexavalent chromium (Cr(VI)) is toxic and can generate ROS through redox reactions, leading to cellular damage	Increased levels of ROS, damage to DNA, lipids, and proteins, increased levels of MDA	

Abbreviations: GSH: glutathione; MDA: malondialdehyde; MnSOD: manganese-dependent superoxide dismutase; ROS: reactive oxygen species.

## Data Availability

No new data were created or analyzed in this study. Data sharing is not applicable to this article.
